# Vaccine hesitancy: the greatest threat to COVID-19 vaccination programs

**DOI:** 10.1186/s42506-021-00081-2

**Published:** 2021-07-05

**Authors:** Samia A. Nossier

**Affiliations:** Family Health Department, High Institute of Public Health, University of Alexandria, Alexandria, Egypt

The availability of an effective vaccine and wide coverage are two crucial factors for the success of vaccination programs. In the early times of the COVID-19 pandemic, the development of an effective vaccine was a mere hope. When this hope turned to be a reality—and one that was realised within an unprecedented time for the development of any other vaccine—, the general population’s response towards receiving the new vaccines was less than optimal. Despite strong evidence that vaccines have proven to be highly effective at preventing both infection and serious illness from COVID-19 [1–3], a high percentage of people still express hesitancy about it [4, 5].

Vaccine hesitancy is defined by the World Health Organization (WHO) as *“*Vaccine hesitancy refers to delay in acceptance or refusal of vaccination despite availability of vaccination services. Vaccine hesitancy is complex and context*-*specific, varying across time, place and vaccines. It is influenced by factors such as complacency, convenience and confidence”. The WHO SAGE Working Group on Vaccine Hesitancy describes hesitancy on a continuum between full acceptance and outright refusal and recognises that hesitance can be to a single or multiple vaccines [6]. The reasons for this behaviour are multifaceted, culture-specific, and often not completely understood [7].

The reluctance or refusal to vaccinate is commonly encountered in almost all vaccination programs. It is usually the result of a combination of factors, such as the perceived risk and severity of infection, confidence in vaccines, values and emotions, as well as environmental and social contexts. COVID-19 vaccine hesitancy has some unique characteristics that are linked to the rapid development of the vaccines, the relatively new techniques used for development of the vaccines, the new mutations of the virus, the occurrence of rare but severe adverse reactions, the need for continued engagement in preventive behaviour even if and after people have been vaccinated, and the diversity and continuous change of policy responses around the world. In addition, being a new disease, the continuous flow of new information about the symptoms, severity, and mortality resulted in confusion and fluctuation in people’s perception of the risks and consequently in uncertainty about the effectiveness of the developed vaccines [8]. Low confidence in vaccination was also heightened by the abundance of misinformation, rumours and false conspiracy theories that circulated in the media [7].

The rates of acceptance and willingness to be vaccinated have varied greatly over the time of the pandemic. Before the availability of COVID-19 vaccines, studies that assessed attitudes of the general public towards vaccines revealed the existence of regional variability with regard to the perception of the safety and effectiveness of vaccination. Higher-income regions were the least certain regarding vaccine safety with 72–73% of people in North America and Northern Europe unsure if vaccines are safe. However, the majority of people in lower-income areas agreed that vaccines are safe. A similar pattern was observed regarding vaccine effectiveness [9].

When more information was available about the process of development of the new vaccines, a lot of misinformation and rumours resulted in lowering the trust of the public in the safety and effectiveness of the vaccine. A systematic review (2021) [10] showed that the Middle East was among the regions with the lowest COVID-19 vaccine acceptance rates globally. Such low rates were most probably related to the widespread beliefs in conspiracy theories in the region. For other parts of the world, the speed at which COVID-19 vaccines were developed and reports of anaphylaxis [11] and blood clots in people receiving the AstraZeneca vaccine in Europe [12] may be causing apprehension that vaccine development was rushed and safety may have been compromised. Public fears increased when some scientists questioned the approval of vaccines for emergency use by the FDA [13]. Social media disseminated a lot of unfounded opinions that adversely affected trust of the vaccine. Posts that circulated widely across the media included, for example, that vaccines have been manufactured to track personal data, are counter to the foundations of the Christian faith, and impact fertility. These worries created scepticism which often affect decision on whether to receive the vaccine [8].

In the early months of 2021, the roll out of vaccines was very high in the USA and the UK and the hesitancy rates have dropped ever since. In the USA, a tracker of vaccine acceptance was used and is regularly updated. As of late April 2021, nearly 100 million people in the USA have been fully vaccinated against COVID-19 and almost 140 million have received at least one dose [14]. While those numbers are positive and indicate that the USA is closer to beating the pandemic, recent data show that 1 in 5 Americans are unwilling to get the COVID-19 vaccine [15]. As more people are successfully vaccinated, trust increases and people realise that social media conspiracy theories are false. Due to the central role that social media plays, it is now assisting the vaccine process in the USA and the UK, rather than hindering it [8].

Wide gaps in vaccine coverage between countries could potentially delay global control of the pandemic. The current levels of willingness to accept a COVID-19 vaccine are insufficient to meet the requirements for herd immunity [16]. In order to inform interventions that improve vaccine coverage, it is imperative to understand the complex and interplaying factors that influence vaccination decisions and the determinants of vaccine hesitancy in a specific population. Research showed that trust in government is strongly associated with vaccine acceptance and can contribute to public compliance with recommended actions [17]. Unfortunately, building trust in vaccine safety and efficacy requires great effort. Programs will not achieve wide coverage unless an in-depth investigation is done to identify the community-specific reasons behind hesitancy. This understanding is essential to inform targeted and context-specific interventions.

To achieve high acceptance and uptake, evidence-based and behaviourally informed strategies should be used. Strategies that are based on traditional informational campaigns aiming to change behaviours by improving knowledge have shown little impact on facilitating vaccination uptake. Research efforts have generated potentially effective strategies. For instance, focusing on building trust in COVID-19 vaccines *before* people form an opinion against them, highlighting the consequences of inaction during consultations with health professionals, and emphasising the social benefits of vaccination. Other strategies—such as reducing barriers, using reminders and planning prompts, and training and building confidence in health workers—have also been shown to be effective [7].
